# Construction and validation of a machine learning‐based nomogram: A tool to predict the risk of getting severe coronavirus disease 2019 (COVID‐19)

**DOI:** 10.1002/iid3.421

**Published:** 2021-03-13

**Authors:** Zhixian Yao, Xinyi Zheng, Zhong Zheng, Ke Wu, Junhua Zheng

**Affiliations:** ^1^ Shanghai Medical Aid Team in Wuhan, Shanghai General Hospital Shanghai Jiao Tong University, School of Medicine Shanghai China; ^2^ Department of Urology, Shanghai General Hospital Shanghai Jiao Tong University School of Medicine Shanghai China; ^3^ Department of Pharmacy, Huashan Hospital Fudan University Shanghai China

**Keywords:** COVID‐19, machine learning, nomogram, severe COVID‐19 prediction

## Abstract

**Background:**

Identifying patients who may develop severe coronavirus disease 2019 (COVID‐19) will facilitate personalized treatment and optimize the distribution of medical resources.

**Methods:**

In this study, 590 COVID‐19 patients during hospitalization were enrolled (Training set: *n* = 285; Internal validation set: *n* = 127; Prospective set: *n* = 178). After filtered by two machine learning methods in the training set, 5 out of 31 clinical features were selected into the model building to predict the risk of developing severe COVID‐19 disease. Multivariate logistic regression was applied to build the prediction nomogram and validated in two different sets. Receiver operating characteristic (ROC) analysis and decision curve analysis (DCA) were used to evaluate its performance.

**Results:**

From 31 potential predictors in the training set, 5 independent predictive factors were identified and included in the risk score: C‐reactive protein (CRP), lactate dehydrogenase (LDH), Age, Charlson/Deyo comorbidity score (CDCS), and erythrocyte sedimentation rate (ESR). Subsequently, we generated the nomogram based on the above features for predicting severe COVID‐19. In the training cohort, the area under curves (AUCs) were 0.822 (95% CI, 0.765–0.875) and the internal validation cohort was 0.762 (95% CI, 0.768–0.844). Further, we validated it in a prospective cohort with the AUCs of 0.705 (95% CI, 0.627–0.778). The internally bootstrapped calibration curve showed favorable consistency between prediction by nomogram and the actual situation. And DCA analysis also conferred high clinical net benefit.

**Conclusion:**

In this study, our predicting model based on five clinical characteristics of COVID‐19 patients will enable clinicians to predict the potential risk of developing critical illness and thus optimize medical management.

## Graphical abstract

We generated the nomogram for predicting severe COVID‐19. (Our predicting model of COVID‐19 patients will enable clinicians to predict the potential risk of developing critical illness and optimize medical management.) In the training cohort, the area under curves (AUCs) were 0.822 (95% CI, 0.765–0.875) and the internal validation cohort was 0.762 (95% CI, 0.768–0.844) and validated it in a prospective cohort with the AUCs of 0.705 (95% CI, 0.627–0.778).

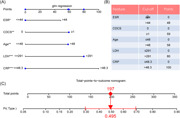

## 1 INTRODUCTION

Severe coronavirus disease 2019 (COVID‐19) outbreaks worldwide during early December 2019. As of May 5, 2020, the number of cumulative cases has surpassed 3,500,000 with over 240,000 deaths worldwide. So far, the global epidemic situation is still very serious. Coronavirus owns distinct immune responses as well as immune escaping features and then creates critical pathogenic processes during inflammation, which subsequently led to lung infection, and edema, acute respiratory distress syndrome (ARDS), or multiple organ dysfunction, and even death.^1^ Therefore, rapid and accurate prediction of COVID‐19 pneumonia trends can provide effective treatment.

As research progresses, more and more information about COVID‐19 pneumonia has been revealed. Lauer et al.^2^ reported that under conservative assumptions, the estimated median incubation period of COVID‐19 is 5.1 days, and in only 101 of every 10,000 cases, symptoms would not develop until after 14 days of active monitoring or isolation. As reported by Huang et al., patients with COVID‐19 present primarily with fever, dry cough, and myalgia or fatigue. Although prognosis of most patients are thought to be favorable, older people and those with weakened immunity may have worse outcomes, even dead.^3^ Through the infection process, sequential evaluation of lymphocyte calculation dynamics and inflammatory contents, including lactate dehydrogenase (LDH), C‐reactive protein (CRP), and interleukin‐6 (IL‐6) may aid to distinguish cases with poor prognosis and prompt medical intervention to increase outcomes.^4^ Zhou et al.^5^ recorded that increased probabilities of in‐hospital mortality are linked to older age (odds ratio 1.10; 95% CI, 1.03–1.17, per year increase; *p* = .0043), which could be the potential risk factor. Another research demonstrated that more grown age, pre‐existed hypertension, raised cytokine levels (IL‐2R, IL‐6, IL‐10, and TNF‐a), and high LDH level were significantly connected to severe COVID‐19 while admitted.^6^ And 1 week after the onset of the disease is a critical period, patients with severe illness may develop dyspnea and hypoxemia within, which may quickly progress to ARDS or end‐organ failure.^7^

Therefore, to identify high‐risk patients whose disease may likely progress is of great importance both in delivering personalized medical care and optimizing medical resource distribution on the macro level. Gong et al.^8^ provided a nomogram to help clinicians to early identify patients who will exacerbate to severe COVID‐19 but they did not take clinical factors like underlying comorbidities in consideration which was a universally acknowledged risk factor. Ji et al.^9^ used the CALL score model (Comorbidity‐Age‐Lymphocyte count‐Lactate dehydrogenase) to estimate the progressive risk of COVID‐19 patients but the sample size was limited, which may cause the volatility of the result, for example, the hazard ratio of LDH > 500 is 9.8 (2.8–33.8).

As a systemic disease, it is necessary to take multiple indicators into account. Comorbidities, age, biochemical indicators (LDH, CRP, and blood urea nitrogen [BUN]), and blood indicators are all potential influencing factors. In this study, we selected 5 effective indexes among 31 items and established an effective 5‐feature based nomogram by machine learning methods. More importantly, to ensure the prediction accuracy, we then further verify this system in a prospective cohort. This could help clinicians to predict the progression of COVID‐19 and provide better‐centralized management.

## 2 MATERIALS AND METHODS

### 2.1 Study population

We retrospectively collected 412 patients from January 1 to February 6, 2020 in Jinyintan Hospital of Wuhan City who were centrally treated and diagnosed with Common or Severe type of COVID‐19. For extra validation, 178 patients were prospectively recruited from February 6, 2020 to March 10, 2020. This study was approved by the Ethics Review Committee of Wuhan Jinyintan Hospital and Shanghai General Hospital.

### 2.2 Diagnostic criteria

According to the “New Coronavirus Pneumonia Diagnosis and Treatment Program (Trial Version 6)” promulgated by the General Office of the National Health Commission^10^ Clinical classification: (1) Light type: mild clinical symptoms, no pneumonia manifestations in imaging; (2) Common type: fever, respiratory tract and other symptoms, pneumonia manifestations can be seen in imaging; (3) Severe type: meet any of the following: ① Respiratory distress, RR ≥ 30 times/min; ② In resting state, it means oxygen saturation ≤ 93%; ③ Arterial blood oxygen partial pressure (PaO2)/oxygen concentration (FiO_2_) ≤ 300 mmHg; (4) Critical Type: Those meet with one of the following conditions: ① have respiratory failure and need mechanical ventilation; ② have shock; ③ combined with other organ failures which require ICU monitoring treatment. More details are provided in the Supporting Information.

### 2.3 Data collection

We collect the hospitalization history of all the subjects and analyze their clinical data, including gender, age, date of onset, time of first diagnosis, time of the definite diagnosis, time of admission, time of discharge, occupation, history of exposure, underlying disease, first symptoms, body signs, laboratory tests, imaging data, and treatment status, etc. Two radiologists were assigned to read the chest radiographs and computerized tomography (CT) of the selected patients, recorded the type of lung lesions and the distribution characteristics of the lung lobes at the time of onset with reached consensus. The New Coronavirus Infection Pneumonia Diagnosis and Treatment Program (Trial Version 6) conducts epidemiological investigations, including whether there is a history of travel or residence in Hubei province and its surrounding areas, whether it has contact with diagnosed patients, and whether there are clustered diseases.

### 2.4 Study design and data processing

Since most of the Light type of COVID‐19 victims do not need medical support or hospitalization and Critical type patients were limited in the hospital, we only analyzed the Common and Severe types, which occupied most of the medical system and equipment.

**Figure 1:**
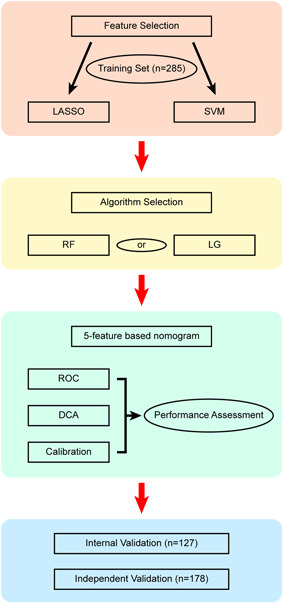
Study flowchart. DCA, decision curve analysis; LASSO, Least Absolute Shrinkage and Selector Operation; LG, logistic regression; SVM‐RFE, Support Vector Machine‐Recursive Feature Elimination; RF, random forest; ROC, receiver operating characteristic

For the research design, we incorporated three sections to identify and validate clinical signature‐based nomograms to predict whether a Common COVID‐19 patient will progress to the Severe type. The study flowchart is shown in Figure 1. Initially, we collected 439 patients and we filtered out 2 Common type, 1 Critical type, and other 24 patients with incomplete medical information. The subsequent 412 patients were divided into the training set (*n* = 285) and internal validation set (*n* = 127) by random seed with a 7:3 ratio. The outcome variables are defined as Critical type = 1 and Common type = 0 in statistical analyses.

For continuous variables, the Maximally Selected Rank Statistics (MSRS) was used to generate the optimal cutoff value and all variables are transformed into dichotomous data.^11^ Following initial filtration, a widely used Least Absolute Shrinkage and Selector Operation (LASSO) method^12^ with the cross‐validation level set at 10‐folds, was built to select suitable traits. Concurrently, we also use the Support Vector Machine‐Recursive Feature Elimination (SVM‐RFE) for hallmarks collection.^13^ Ultimately, we intersect clinical features from the LASSO and the SVM‐RFE methods and subsequently use the multivariate logistic regression (LG) and random forest (RF) to test the predictive power of the model. Superior to random forest performance, multivariate logistic regression was used to build the predictive nomogram in 285 patients and internally validated in 127 patients. In the independent validation phase, candidate features were validated in a prospective cohort (*n* = 178) (Figure 1).

### 2.5 Statistical analysis

To operate 10‐fold cross‐validated LASSO and 5‐fold cross‐validated SVM‐RFE algorithm, we applied *glmnet* and *e1071* in R language, respectively. The random forest algorithm was realized by R package *randomForest* and the number decision‐making tree was set to 1000. To measure the ability of the nomogram in the validation data, we employ the concordance index and the calibration plots to investigate the graphical performance where the R package *rms* was applied. And to enhance its clinical application, the predictive display of the nomogram was estimated by the analysis of receiver operating characteristic (ROC) as well as area under the curve (AUC) conditions. Decision curve analysis (DCA) were performed to plot net benefit (NB) as well as cost versus benefit ratio and assess the utility of models for decision making.^14^ In risk assessment tools, the predicted probability that a patient is diagnosed with certain disease is recorded as *P*_*i*_; when *P_i_* reaches a certain threshold (denoted as *P*_t_), it is defined positive, which is Severe COVID‐19 in our study. And the formula is as follows:

$$\begin{array}{lll} \text{NB} & = & \text{True}\;\text{positive}\;\text{rate}\;–\;(\text{False}\;\text{positive}\;\text{rate}\\ & & \times \;\text{Weighting}\;\text{factor}),\text{Weighting}\;\text{factor}\\ & = & P_{t}/(1-P_{t}),\text{Cost}:\text{Benefit}\;\text{Ratio}\\ & = & \text{True}\;\text{positive}\;\text{cases}/\text{False}\;\text{positive}\;\text{cases}. \end{array}$$

Hosmer–Lemeshow goodness‐of‐fit test is used to validate the model fitness.^15^ Statistical investigations were conducted in the 3.6.1 version of R language and *p* < .05 (both sides) is thought to be statistically significant.

## 3 RESULTS

### 3.1 Patient characteristics

Taken together, 590 confirmed cases with COVID‐19 were recruited from February 1st through March 10th. Two hundred twenty‐six (38.5%) of them developed severe diseases during hospitalization and the median hospitalization time is 12 days. The optimal cutoff value and the distribution of patients' characteristics in three cohorts are presented in Table 1.

**Table 1:** Baseline characteristics of COVID‐19 patients

	Training set (%)	Internal validation (%)	Prospective validation (%)	
	*N* = 285	*N* = 127	*N* = 178	*p* value
Type				
Common	184 (64.6)	81 (63.8)	98 (55.1)	.104
Severe	101 (35.4)	46 (36.2)	80 (44.9)	
Gender				
Male	152 (53.3)	64 (50.4)	92 (51.7)	.847
Female	133 (46.7)	63 (49.6)	86 (48.3)	
Age				
≤48	136 (47.7)	70 (55.1)	78 (43.8)	.148
>48	149 (52.3)	57 (44.9)	100 (56.2)	
CDCS				
≥1	82 (28.8)	33 (26.0)	53 (29.8)	.761
0	203 (71.2)	94 (74.0)	125 (70.2)	
WBC (10^9^/L)				
≤9.16	250 (87.7)	108 (85.0)	153 (86.0)	.727
>9.16	35 (12.3)	19 (15.0)	25 (14.0)	
Hb (g/L)				
≤133	189 (66.3)	88 (69.3)	122 (68.5)	.798
>133	96 (33.7)	39 (30.7)	56 (31.5)	
PLT (10^9^/L)				
≤155	67 (23.5)	26 (20.5)	49 (27.5)	.348
>155	218 (76.5)	101 (79.5)	129 (72.5)	
Lymphcyte (10^9^/L)				
≤0.8	81 (28.4)	32 (25.2)	46 (25.8)	.733
>0.8	204 (71.6)	95 (74.8)	132 (74.2)	
ESR (mm/h)				
≤44	99 (34.7)	46 (36.2)	69 (38.8)	.681
>44	186 (65.3)	81 (63.8)	109 (61.2)	
CRP (mg/L)				
≤48.3	196 (68.8)	94 (74.0)	123 (69.1)	.536
>48.3	89 (31.2)	33 (26.0)	55 (30.9)	
TBIL (μmol/L)				
≤8.2	47 (16.5)	23 (18.1)	35 (19.7)	.682
>8.2	238 (83.5)	104 (81.9)	143 (80.3)	
DBIL (μmol/L)				
≤2.9	91 (31.9)	29 (22.8)	43 (24.2)	.075
>2.9	194 (68.1)	98 (77.2)	135 (75.8)	
ALT (μ/L)				
≤14	37 (13.0)	25 (19.7)	24 (13.5)	.181
>14	248 (87.0)	102 (80.3)	154 (86.5)	
AST (μ/L)				
≤21	58 (20.4)	22 (17.3)	23 (12.9)	.123
>21	227 (79.6)	105 (82.7)	155 (87.1)	
ALB (g/L)				
≤31.7	122 (42.8)	55 (43.3)	81 (45.5)	.845
>31.7	163 (57.2)	72 (56.7)	97 (54.5)	
GLB (g/L)				
≤28.9	70 (24.6)	33 (26.0)	56 (31.5)	.256
>28.9	215 (75.4)	94 (74.0)	122 (68.5)	
BUN (mmol/L)				
≤4.48	159 (55.8)	63 (49.6)	88 (49.4)	.312
>4.48	126 (44.2)	64 (50.4)	90 (50.6)	
Cr (μmol/L)				
≤71.5	169 (59.3)	79 (62.2)	103 (57.9)	.745
>71.5	116 (40.7)	48 (37.8)	75 (42.1)	
GLU (mmol/L)				
≤6.9	208 (73.0)	88 (69.3)	124 (69.7)	.647
>6.9	77 (27.0)	39 (30.7)	54 (30.3)	
CK (μ/L)				
≤106	214 (75.1)	100 (78.7)	125 (70.2)	.228
>106	71 (24.9)	27 (21.3)	53 (29.8)	
CK‐MB (μ/L)				
≤11.1	100 (35.1)	43 (33.9)	54 (30.3)	.569
>11.1	185 (64.9)	84 (66.1)	124 (69.7)	
LDH (μ/L)				
≤291	174 (61.1)	85 (66.9)	106 (59.6)	.394
>291	111 (38.9)	42 (33.1)	72 (40.4)	
K^+^ (mmol/L)				
≤4.2	154 (54.0)	70 (55.1)	94 (52.8)	.922
>4.2	131 (46.0)	57 (44.9)	84 (47.2)	
Ca^+^ (mmol/L)				
≤1.99	120 (42.1)	50 (39.4)	79 (44.4)	.682
>1.99	165 (57.9)	77 (60.6)	99 (55.6)	
AP (mg/L)				
≤127.5	123 (43.2)	61 (48.0)	70 (39.3)	.318
>127.5	162 (56.8)	66 (52.0)	108 (60.7)	
Mb (ng/ml)				
≤92.4	253 (88.8)	118 (92.9)	153 (86.0)	.164
>92.4	32 (11.2)	9 (7.1)	25 (14.0)	
Tn (pg/ml)				
≤0.9	72 (25.3)	38 (29.9)	46 (25.8)	.598
>0.9	213 (74.7)	89 (70.1)	132 (74.2)	
PCT (ng/ml)				
≤0.069	222 (77.9)	102 (80.3)	145 (81.5)	.631
>0.069	63 (22.1)	25 (19.7)	33 (18.5)	
D‐dimer (μg/ml)				
≤3.03	260 (91.2)	115 (90.6)	156 (87.6)	.445
>3.03	25 (8.8)	12 (9.4)	22 (12.4)	
IL‐6 (pg/ml)				
≤8.17	183 (64.2)	91 (71.7)	126 (70.8)	.195
>8.17	102 (35.8)	36 (28.3)	52 (29.2)	
AMY (U/L)				
≤61.2	136 (47.7)	58 (45.7)	82 (46.1)	.905
>61.2	149 (52.3)	69 (54.3)	96 (53.9)	
LPS (U/L)				
≤29	92 (32.3)	31 (24.4)	50 (28.1)	.245
>29	193 (67.7)	96 (75.6)	128 (71.9)	
FER (ng/ml)				
≤273.54	82 (28.8)	35 (27.6)	33 (18.5)	.04
>273.54	203 (71.2)	92 (72.4)	145 (81.5)	

*Note: p* value was calculated by Fisher's exact test.

Abbreviations: ALB, albumin; ALT, alanine transaminase; AMY, amylase; AP, amyloid protein; AST, glutamic oxalacetic transaminase; BUN, blood urea nitrogen; CDCS, Charlson/Deyo comorbidity score; CK, creatine kinase; CK‐MB, creatine kinase isoenzyme‐MB; COVID‐19, coronavirus disease 2019; Cr, creatinine; CRP, C‐reactive protein; DBIL, direct bilirubin; ESR, erythrocyte sedimentation rate; FER, ferritin; GLB, globulin; GLU, glucose; Hb, hemoglobin; IL‐6, interleukin‐6; LDH, lactate dehydrogenase; LPS, lipase; Mb, myohemoglobin; PCT, procalcitonin; PLT, platelet; TBIL, total bilirubin; Tn, troponin; WBC, white blood cell.

### 3.2 Selection of candidate clinical features

**Figure 2:**
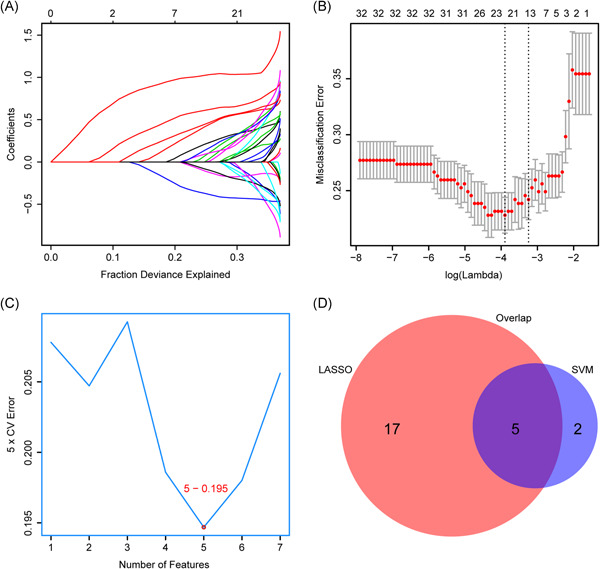
Two algorithms were used for feature selection. (A) Profiles of coefficients with penalization were plotted against the log (lambda) sequence, which explains the fraction deviance. (B) Tenfold cross‐validated error (first vertical line equals the minimum error (lambda = 0.033) and the second one shows the cross‐validated error within 1 standard error of the minimum) in LASSO. (C) Fivefold cross‐validated SVM‐RFE algorithms in the training cohort. (D) The intersection of important features. LASSO, Least Absolute Shrinkage and Selector Operation; SVM‐RFE, Support Vector Machine‐Recursive Feature Elimination

**Figure 3:**
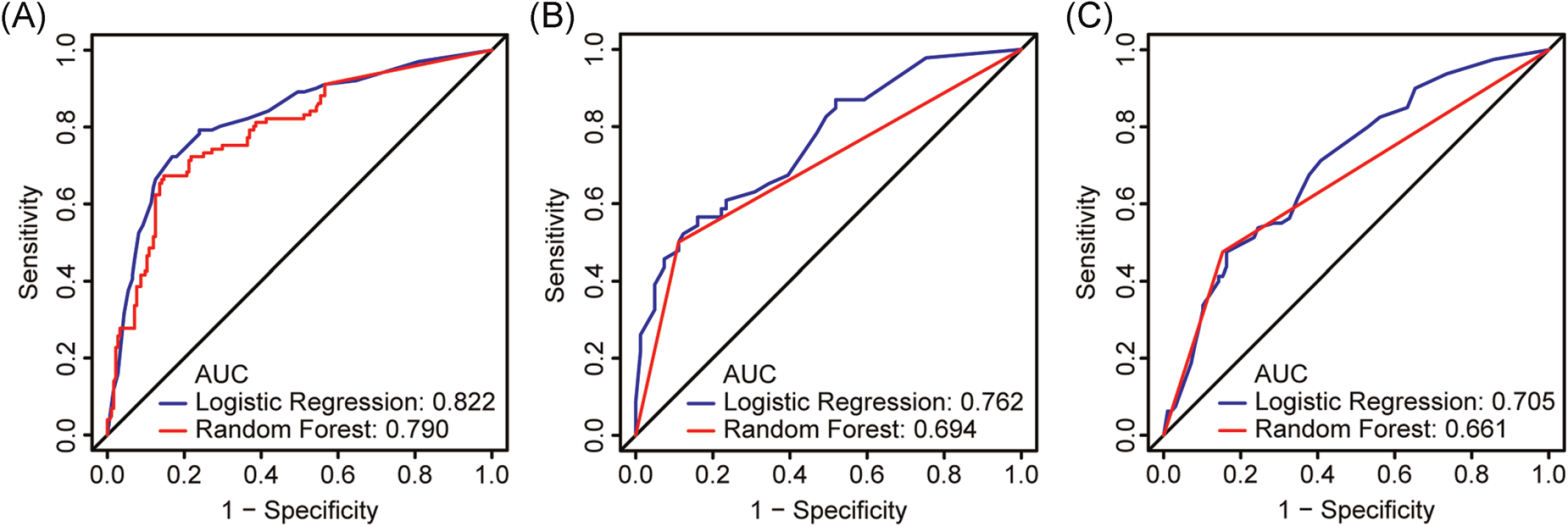
ROC analysis to assess the performance of LG and RF in predicting the severe type COVID‐19 in the three cohorts. (A) Training cohort, (B) internal validation cohort, and (C) external validation cohort. AUC, area under the curve; COVID‐19, coronavirus disease 2019; LG, logistic regression; RF, random forest; ROC, receiver operating characteristic

Accepting the initial filter standards specified in the Methods segment, we used 10‐fold cross‐validated LASSO method and 5‐fold cross‐validated SVM‐RFE (Figure 2A–C), two different machine learning algorithms and derived five clinical features, which are ESR, CDCS, Age, LDH, and CRP (Figure 2D). The cutoff value and the distribution of primary continuous variables were showed in Figure S1. Next, we used LG and RF to build the predictive model based on the five selected features (Figure 3). And the LG model demonstrated better AUCs among all the data sets than the RF model, and thus were chosen for subsequent analysis. Subsequent multivariate logistic analysis revealed that ESR > 44 mm/h (HR, 1.97; 95% CI, 1.03–3.89; *p* = .04), CDCS ≥ 1 (HR, 2.31; 95% CI, 1.23–4.35; *p* = .009), age > 48 years (HR, 2.27; 95% CI, 1.24–4.19; *p* = .008), LDH > 291 U/L (HR, 3.08; 95% CI, 1.7–5.61; *p* < .001), and CRP > 48.3 mg/L (HR, 4.1; 95% CI, 2.23–7.62; *p* < .001) were critical risk factors closely related to the progression of COVID‐19 (Table 2).

**Table 2:** Univariate and multivariate logistic regression analysis of progression of COVID‐19 patients in training cohort

	Univariate logistic analysis		Multivariate logistic analysis	
	OR (95% CI)	*p* value	OR (95% CI)	*p* value
ESR (mm/h)				
≤44	1	–	1	–
>44	3.05 (1.75–5.49)	<.001	1.97 (1.03–3.89)	.04
CDCS				
0	1	–	1	–
≥1	2.97 (1.75–5.07)	<.001	2.31 (1.23–4.35)	.009
Age				
≤48	1	–	1	–
>48	3.7 (2.21–6.33)	<.001	2.27 (1.24–4.19)	.008
LDH (μ/L)				
≤291	1	–	1	–
>291	4.7 (2.81–7.97)	<.001	3.08 (1.7–5.61)	<.001
CRP (mg/L)				
≤48.3	1	–	1	–
>48.3	7.21(4.17–12.73)	<.001	4.1 (2.23–7.62)	<.001

Abbreviations: CDCS, Charlson/Deyo comorbidity score; CI, confidence interval; CRP, C‐reactive protein; COVID‐19, coronavirus disease 2019; ESR, erythrocyte sedimentation rate; LDH, lactate dehydrogenase; OR, odds ratio.

### 3.3 Building a predictive signature

**Figure 4:**
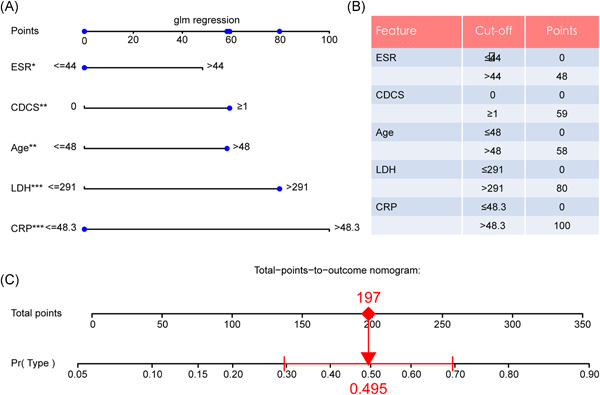
Predictive model and feature risk scores. (A) Nomogram to predict the severe type COVID‐19. (B) Risk scores for each feature. (*p < .05, **p < .01, ***p < .001). CDCS, Charlson/Deyo comorbidity score; COVID‐19, coronavirus disease 2019; CRP, C‐reactive protein; ESR, erythrocyte sedimentation rate; LDH, lactate dehydrogenase

To develop a clinically applicable tool that could predict the probability of whether a COVID‐19 patient can develop severe disease, we constructed a nomogram to develop a predictive model, considering clinical covariates (Figure 4A). The predictors included CRP, LDH, Age, CDCS, and ESR and the risk score of each covariate produced by the LG model are listed (Figure 4B). To select one subject for instance (blue track in Figure 4A), based on the chosen traits, the cumulative scores add up to 197 and hence the probabilities of progressing to severe COVID‐19 is 0.495.

### 3.4 Validating and assessing the model

To complete the steadiness of the nomogram, validation judgments were operated in an internal validation set (*n* = 127) as well as a prospective validation cohort (*n* = 178). ROC analysis revealed that the nomogram exhibited alike AUC values of 0.822 (95% CI, 0.765–0.875; *p* < .001), 0.762 (95% CI, 0.768–0.844; *p* < .001), and 0.705 (95% CI 0.627–0.778; *p* < .001) for the evaluation of potential severe COVID‐19 cases (Figure 3A–C).

**Figure 5:**
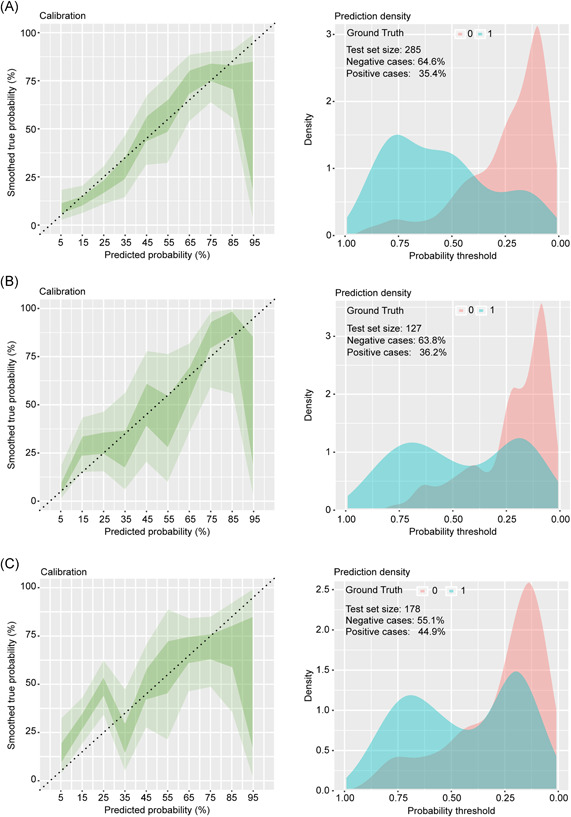
Calibration curve and prediction density plot for nomogram model. (A,D) Training cohort, (B,E) internal validation cohort, (C,F) prospective validation cohort. The dotted line represents the ideal nomogram, and the deep green track represents the observed nomogram with the diluted green track representing confidence interval

**Figure 6:**
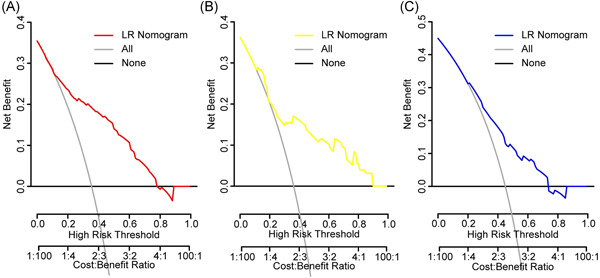
Decision curve analysis for nomogram. The black transverse line represents none model conditions. Gray curves are on behalf of all cases developing severe COVID‐19. The first abscissa axis represents the threshold probability which means the probability of developing severe COVID‐19 and the second represents the cost versus benefit ratio. And the vertical axis stands for the net benefit of the nomogram. (A) Training cohort, (B) internal validation cohort, and (C) prospective validation cohort. COVID‐19, coronavirus disease 2019; LR, likelihood ratio

**Figure 7:**
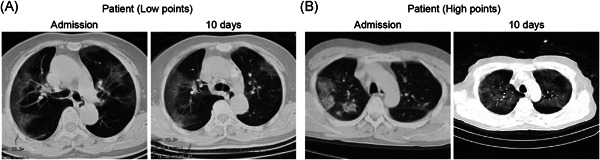
Two example CT scans of victims with distinguished risk points. Cross‐section CT scans of initial admission and 10 days after of two patients. (A) Low points patient and (B) high points patient. CT, computerized tomography

The bootstrapped calibration plot for the probability showed consistency amid forecast by nomogram and original observation (Figure 5A–C) and favorable discriminative power (Figure 5D–F). And Hosmer–Lemeshow test *p* value are .501 (training set), .239 (internal validation set), and .453 (prospective validation set), which indicate the good fitness of the model. And the DCA plots all show the favorable net benefit for clinical use compared to none model conditions (Figure 6A–C). To take two patients with distinguished risk scores, for example, the low‐risk one added up to 74 points with the probability of 0.12 to progress and the CT scan showed no worsen pneumonia after a 10‐day hospitalization. Another one added up to 361 points (high risk) with the probability of 0.89 went through severe lung lesion in 10 days (Figure 7A,B), which then developed permanent lung damage.

## 4 DISCUSSION

Since the COVID‐19 broke out in Hubei, even with the optimal control in China, the cumulative confirmed cases in the globe had overpassed three million and the threat of coronavirus is still out there. In the patients who suffered COVID‐19, CRP was increased in 86.22% of them, and ESR in 90.22%.^16^ Until now, more and more independent risk factors have been determined and a number of systemic score systems have been built to analyze the status of disease progression and prevent severe outcomes. Ji et al.^9^ reported a novel scoring model, named CALL, established for disease condition prediction, which included comorbidity, age, lymphocyte, and LDH, with the AUC reaching 0.91 (95% CI, 0.86–0.94). However, only one validation of this model has been made, the robustness of it calls for further justification, especially considering the sample size in some subgroups are fairly small, like LDH. Another model to early predict severe type of COVID‐19 showed older age, higher LDH, CRP, RDW, DBIL, BUN, and lower ALB on admission correlated with higher odds of severe COVID‐19, with the AUC reached 0.912 (95% CI, 0.846–0.978) in the training set, and 0.853 (95% CI, 0.790–0.916) in the validation set.^8^ Despite the relatively limited sample size, too many continuous variables enrolled can cause inconvenience in real‐world implementation.

For the sake of controlling the major health incident and bettering the medical resource allocation, we extracted the clinical data of 590 cases from the Wuhan Jinyintan Hospital and the prediction model has been established, which included ESR, CDCS, Age, LDH, and CRP. Notably, CDCS, the Charlson/Deyo comorbidity score, a system of classifying comorbidities of victims referring to the International Classification of Diseases (ICD) diagnosis codes found in organizational data, such as hospital abstracts data. Each comorbidity classification has a corresponding measurement (from 1 to 6), based on the adjusted risk of mortality or resource use, and the whole weights add up to a single comorbidity score for each victim. A sum of zero means that no comorbidities are found. If the score gets higher, it means the patient is more likely to develop a poorer outcome. These scoring systems have been reported to be associated with overall survival in various types of cancer and the death rate of other morbidities, such as ischemic stroke, acute cholecystitis, acute hip fracture, and so forth.^17^, ^18^, ^19^, ^20^, ^21^ Due to the efficacy of CDCS, we groundbreakingly utilized this scoring system in our prediction model with meeting the rule of the TRIPOD Statement,^22^ which could also interpret the high mortality of COVID‐19 with multiple comorbidities. Hereby, the five significant indices were overlapped by the LASSO and SVM analysis, which are machine learning used for classification and regression analysis to enhance the prediction accuracy and interpretability of the statistical model it produces. Then, the ROC, DCA, and Calibration analysis were performed for performance assessment, and the triple verification were applied. The predictive nomogram indicated that the possibility of the progression from common type to severe type could reach 50%, when the total points meet 197. Thereafter, the AUC of the internal training set, testing set, and external testing set reached 0.822, 0.762, and 0.705, respectively.

However, there are still some limitation that should be majorized in the future investigation. The AUC values are lower than 0.9 and more cases should be recruited to optimize our prediction model for more precise forecasting. And the data of patients were derived from Wuhan, Hubei Province, which means the situation outside Hubei Province could be distinct and multicenter analysis is urgently needed.

## 5 CONCLUSION

Through the filtering by LASSO and SVM‐RFE, two machine learning methods, five independent predictive features for severe COVID‐19 were selected, which are: CRP, LDH, Age, CDCS, and ESR. Based on this, we build a predicting tool that can early predict severe COVID‐19 and aid medical decisions for COVID‐19 patients.

## CONFLICT OF INTERESTS

The authors declare that there are no conflict of interests.

## AUTHOR CONTRIBUTIONS

Junhua Zheng formulated the original concept and designed the investigation. Ke Wu, Zhong Zheng, and Zhixian Yao engaged in designing the research as well as in the data extraction. Xinyi Zheng and Zhixian Yao examined the data and drafted the paper. All authors read and approved the ultimate document.

## ETHICS STATEMENT

This study was approved by the Ethics Review Committee of Wuhan Jinyintan Hospital and Shanghai General Hospital.

## Supporting information

Supporting information.

Supporting information.
