# Supplementary figures and images for: Construction and validation of a machine learning‐based nomogram: A tool to predict the risk of getting severe coronavirus disease 2019 (COVID‐19)
Source: Immun Inflamm Dis. 2021 Mar 13;9(2):595–607. doi: 10.1002/iid3.421 (PMC8127556; doi:10.1002/iid3.421)

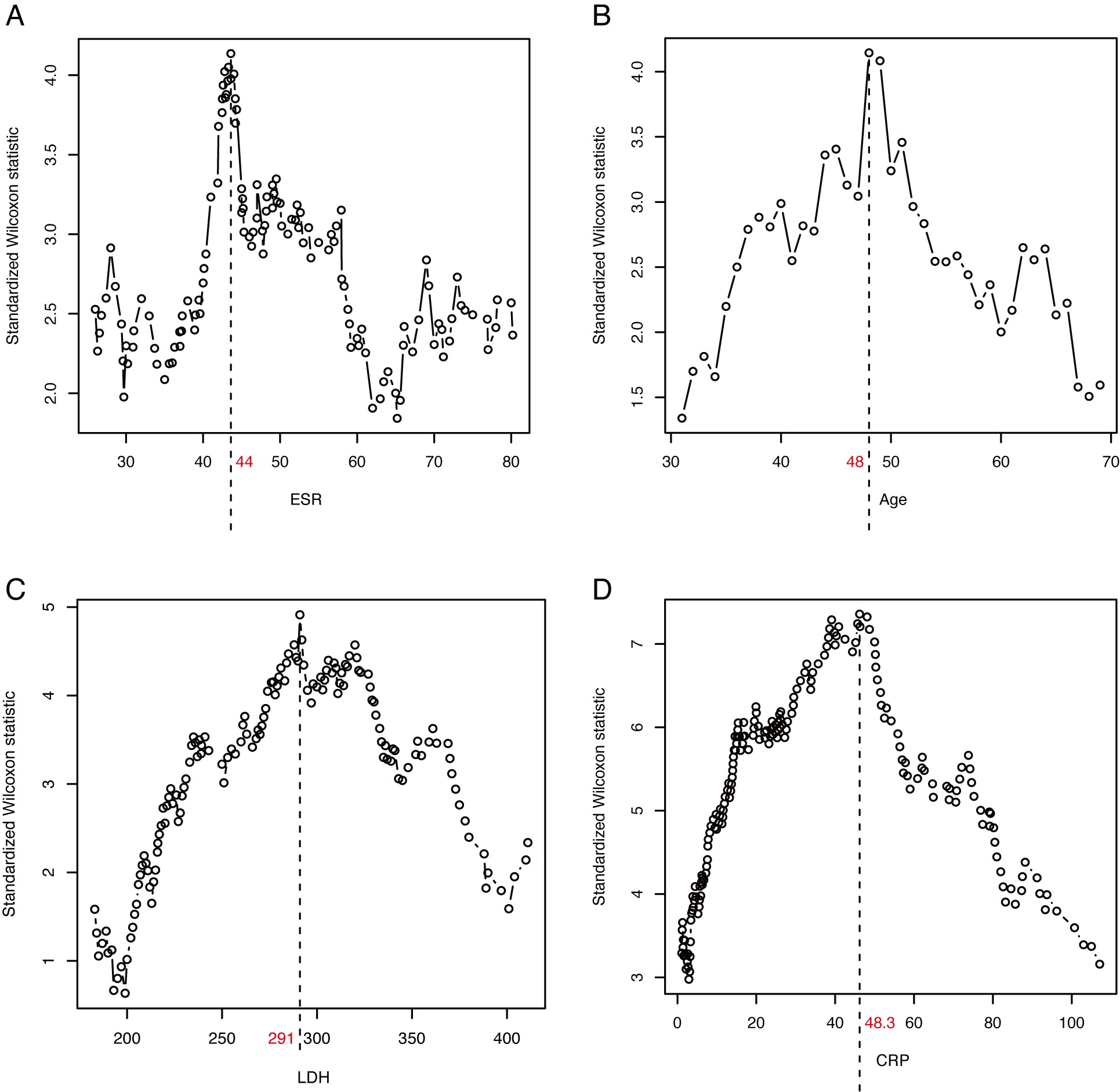

Supplement: Supplementary file 1 — Supporting information. [file IID3-9-595-s002.tif]
