# Supplementary material for: Construction and validation of a machine learning‐based nomogram: A tool to predict the risk of getting severe coronavirus disease 2019 (COVID‐19)
Source: Immun Inflamm Dis. 2021 Mar 13;9(2):595–607. doi: 10.1002/iid3.421 (PMC8127556; doi:10.1002/iid3.421)
Supplement: Supplementary file 2 — Supporting information. [file IID3-9-595-s001.docx]

Diagnostic criteria:

According to the "New Coronavirus Pneumonia Diagnosis and Treatment Program (Trial Version 6)"

Epidemiological history: ①Have a history of travel or residence in Wuhan City and surrounding areas, or other areas where cases have been reported, within 14 days before the onset of illness; Have a history of contact; ③have been exposed to patients with fever or respiratory symptoms from Wuhan and surrounding areas, or from communities with reported cases within 14 days before the onset of illness; Respiratory symptoms; ② Imaging features of the new coronavirus pneumonia; ③ Normal or decreased white blood cells or decreased lymphocyte count in the early stage of onset; (3) Etiological evidence: ① Real-time fluorescent RT-PCR of respiratory tract specimens or blood specimens to detect new coronavirus nucleic acids Positive; ②Sequencing of viral genes in respiratory specimens or blood specimens is highly homologous to known new coronaviruses. Any one of the history of epidemiology or no history of epidemiology, at the same time meet any 2 of the clinical manifestations, plus 1 evidence of etiology can be diagnosed.
